# Assessing nutritional pigment content of green and red leafy vegetables by image analysis: Catching the “red herring” of plant digital color processing via machine learning

**DOI:** 10.1093/biomethods/bpaf027

**Published:** 2025-04-09

**Authors:** Avinash Agarwal, Filipe de Jesus Colwell, Viviana Andrea Correa Galvis, Tom R Hill, Neil Boonham, Ankush Prashar

**Affiliations:** School of Natural and Environmental Sciences, Newcastle University, Newcastle upon Tyne, UK; Institute for Bio- and Geosciences: Plant Sciences (IBG-2), Forschungszentrum Jülich GmbH, Jülich, Germany; Crop Science R&D Division, InFarm—Indoor Urban Farming B.V., Amsterdam, The Netherlands; Crop Science R&D Division, InFarm—Indoor Urban Farming B.V., Amsterdam, The Netherlands; Faculty of Medical Sciences, Newcastle University, Newcastle upon Tyne, United Kingdom; School of Natural and Environmental Sciences, Newcastle University, Newcastle upon Tyne, UK; School of Natural and Environmental Sciences, Newcastle University, Newcastle upon Tyne, UK

**Keywords:** image analysis, machine learning, chlorophyll, carotenoid, anthocyanin, nutrition

## Abstract

Estimating pigment content of leafy vegetables via digital image analysis is a reliable method for high-throughput assessment of their nutritional value. However, the current leaf color analysis models developed using green-leaved plants fail to perform reliably while analyzing images of anthocyanin (Anth)-rich red-leaved varieties due to misleading or “red herring” trends. Hence, the present study explores the potential for machine learning (ML)-based estimation of nutritional pigment content for green and red leafy vegetables simultaneously using digital color features. For this, images of *n *=* *320 samples from six types of leafy vegetables with varying pigment profiles were acquired using a smartphone camera, followed by extract-based estimation of chlorophyll (Chl), carotenoid (Car), and Anth. Subsequently, three ML methods, namely, Partial Least Squares Regression (PLSR), Support Vector Regression (SVR), and Random Forest Regression (RFR), were tested for predicting pigment contents using RGB (Red, Green, Blue), HSV (Hue, Saturation, Value), and L*a*b* (Lightness, Redness-greenness, Yellowness-blueness) datasets individually and in combination. Chl and Car contents were predicted most accurately using the combined colorimetric dataset via SVR (*R^2^* = 0.738) and RFR (*R^2^* = 0.573), respectively. Conversely, Anth content was predicted most accurately using SVR with HSV data (*R^2^* = 0.818). While Chl and Car could be predicted reliably for green-leaved and Anth-rich samples, Anth could be estimated accurately only for Anth-rich samples due to Anth masking by Chl in green-leaved samples. Thus, the present findings demonstrate the scope of implementing ML-based leaf color analysis for assessing the nutritional pigment content of red and green leafy vegetables in tandem.

## Introduction

Traditionally, cultivation has been focused on producing high-biomass crops such as grains, fruits, and tubers, whereas leafy crops were mostly considered a supplement. However, in recent years leafy vegetables have been recognized as a “superfood” owing to them being a source of numerous nutritional substances such as antioxidants and minerals, as well as dietary fibers that promote gut health [1–3]. Amongst these beneficial dietary phytoconstituents, chlorophylls (Chl), carotenoids (Car), and anthocyanins (Anth) are three nutritional pigments well-known to have a positive impact on human health [4–7].

While Chl and Car are abundantly present in numerous green-leaved crops, focus on producing Anth-rich red-leaved crops has intensified in the past decade owing to growing awareness regarding the potential health benefits of Anth [5]. Consequently, there have been concerted efforts to promote large-scale production of various Anth-rich leafy vegetables belonging to diverse plant families, including Amaranthaceae, Brassicaceae, and Lamiaceae [8]. This interest in large-scale cultivation of Anth-rich vegetables has brought to light a new challenge for growers: large-scale assessment of the nutritional quality of such crops in a cost-effective and rapid manner.

In the current scenario, machine vision has become a standard tool for high-throughput, noninvasive assessment of crop health and nutritional quality [9–13]. Amongst the different machine vision technologies being used for large-scale crop monitoring, digital cameras stand out as the most widely used due to their affordability, ease of application, and the strong connection between leaf pigmentation and digital color features [14–18]. These digital color features, primarily recorded in terms of Red–Green–Blue (RGB) reflectance, can be easily translated to other three-dimensional color spaces, such as Hue–Saturation–Value (HSV) and Lightness–Redness-greenness–Yellowness-blueness (L*a*b*), enabling a more in-depth assessment of plant digital color profile. In addition, compatibility of these digitized colorimetric features with modern analytical tools such as machine learning (ML) allows the implementation of highly advanced data processing approaches for assessing crop quality more accurately [19–24].

Notably, a majority of protocols for estimating leaf pigment contents using digital color features have been developed using green-leaved plants due to the prevalence of such crops in conventional commercial cultivation, the primary focus being Chl and Car estimation [25–29]. In contrast, only a few studies have been carried out with red-leaved Anth-rich plants for estimating Anth content [30, 31]. Interestingly, co-estimation of all three types of pigments simultaneously across multiple plant species via generalized models remains largely unexplored, possibly owing to misleading or “red herring” shifts in digital color features in the presence of high Anth concentrations.

Hence, the current study aims to assess the feasibility of estimating Chl, Car, and Anth contents in green- and red-leaved crops concurrently by using ML to process digital color features and generate generalized multi-species models. For this, samples from six different leafy vegetables with varying nutritional pigment profiles were photographed digitally. Color features of the leaf samples were used to generate fundamental and advanced ML-based regression models for noninvasive high-throughput quantification of these three pigments simultaneously across multiple crop species, including both anthocyanic and nonanthocyanic varieties. Subsequently, impact of leaf Anth content on the best-performing prediction models was also assessed.

## Materials and methods

### Plant material

**Figure 1:**
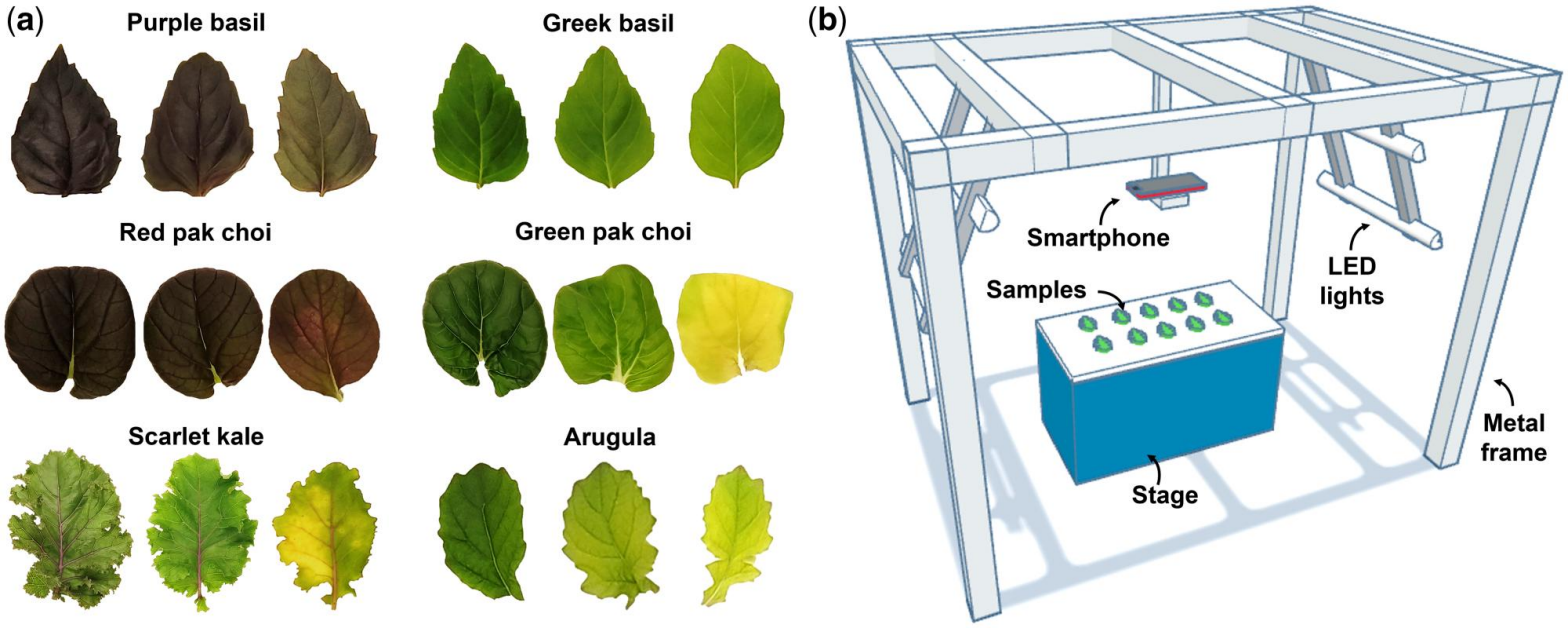
Variations in pigmentation across leaf samples from the six leafy vegetables used in the present study (a), and a schematic overview of the image acquisition setup (b)

The study was carried out with six commercially available leafy vegetables (Fig. 1a), namely, purple basil (*Ocimum basilicum* L. var. *purpurascens*; PB), Greek basil (*Ocimum basilicum* L. var. *minimum*; GB), red pak choi (*Brassica rapa* L. ssp. *chinensis* cv. “Rubi F1”; RPC), green pak choi (*Brassica rapa* L. ssp. *chinensis*; GPC), scarlet kale (*Brassica oleracea* L. var. *acephala* “Scarlet”; SK), and arugula (*Eruca vesicaria* ssp. *sativa* Mill. cv. ‘Wasabi Rocket’; WR). The red leafy vegetables (RLV), i.e. PB and RPC, had Anth-rich dark purple and reddish-green leaves. In contrast, the green leafy vegetables (GLV), i.e. GB, GPC, and WR, displayed various shades of green with no hint of red. SK possessed green leaves with a reddish-tinge and prominent red midrib and veins, and was hence designated as the red-green leafy vegetable (RGLV).

Seedlings of all six leafy vegetables were initiated in coco-peat plugs within a nursery (Aralab, InFarm UK Ltd., London, UK), at a density of 5–10 seedlings per plug. Upon reaching a height of approximately 5 cm, the seedlings were transferred to an experimental hydroponic vertical farm (InStore Farm V2, InFarm UK Ltd.) located at the Agriculture Building, Newcastle University, UK. A total of 24 seedling plugs were taken for each type of leafy vegetable and distributed across two hydroponic trays (dimensions: 30 × 40 cm^2^ each). A commercially-available hydroponics fertilizer mix was used as the nutrient source, and the ebb-and-flow method was implemented for flooding the hydroponic chamber with nutrient solution at regular intervals (10 min/h). White LEDs with approximate red (400–499 nm): green (500–599 nm): blue (600–699 nm) distribution of 40:20:40 were used to maintain a PPFD of 280 µmol/m^2^ s following a 16/8 h day–night cycle. Growth conditions were maintained at 25 ± 1°C and 65 ± 5% relative humidity via a custom-made HVAC system [32]. Plant growth environment was monitored using sensors for temperature, humidity, flow rate, electrical conductivity, and pH via a Farmboard (InFarm UK Ltd.).

### Leaf sampling and image acquisition

Leaf sampling was done at 15–20 days of growth within the hydroponics chamber. Fully expanded leaves with diverse levels of pigmentation (Fig. 1a) were selectively excised at the base and immediately transferred to a customized setup for imaging (Fig. 1b). The setup included a frame for mounting a smartphone, four neutral-white (4000 K) LED tube-lights (Model No. 0051048, Feilo Sylvania International Group Kft., Budapest, Hungary; www.sylvania-lighting.com) for steady lighting, as well as a stage with a white matte surface for placing the leaf samples. A Redmi Note 7 Pro smartphone (Xiaomi Corp., Beijing, China) having Sony IMX 586 RGB sensor (size 1/2.0”, Quad-Bayer array) with a dual rear-camera system (primary lens: resolution 48 megapixels, aperture *f*/1.8, wide angle, pixel size 1.6 µm, phase detection autofocus; secondary lens: resolution 5 megapixels, aperture *f*/2.4, depth perception) was used for image acquisition. The images (8000 × 6000 pixels, sRGB color space, JPEG format) were captured using the Open Camera android application (ver. 1.52, developer: Mark Harman, source: Google Play Store). A distance of 50 cm was maintained between the camera and stage, along with fixed exposure time 1/100 s and ISO-200; automatic adjustments such as auto-focus and exposure compensation were disabled.

### Destructive quantification of pigment contents

Chl, Car, and Anth contents were evaluated spectrophotometrically following image acquisition. Briefly, two sections (2 cm^2^ each) were excised from each leaf, weighed individually, sealed into separate vials, and transferred to −20°C for storage. The sections were subsequently put in a liquid nitrogen bath and pulverized with stainless-steel beads using a tissue homogenizer (Geno/Grinder 2010, SPEX SamplePrep, Cole-Parmer, Illinois, USA). One batch of vials was used for quantifying Chl and Car contents, and the other batch for Anth content.

Chl and Car contents were assessed as described by Lichtenthaler [33]. Briefly, each vial was added with chilled 80% (v/v) acetone (1 mL) and vortexed, followed by centrifugation at 10 000*g* at 4°C for 15 min. The supernatant was collected, and the tissue-pellet was re-washed with 1 mL of the solvent. Both extracts were pooled, and absorbance was recorded at 470 nm (A_470_), 647 nm (A_647_), and 663 nm (A_663_). Total Chl and Car contents were calculated per unit leaf fresh weight (FW) for unit volume (V) of extract as follows:

(1) $$\mathrm{Chl}\;(\frac{\mathrm{mg}}{g\;\mathrm{FW}})=\frac{(18.71A_{647}+7.15A_{663})\;\times \;V}{1000\;\times \;\mathrm{FW}}\;$$

 

(2) $$\mathrm{Car}\;(\frac{\mathrm{mg}}{g\;\mathrm{FW}})=\frac{(1000A_{470}-1822.85A_{647}+411.31A_{663})\;\times \;V}{198\;\times \;1000\;\times \;\mathrm{FW}}\;$$

A similar extraction procedure as above was followed for Anth using chilled acidified (1% w/v HCl) methanol as the solvent [34]. Absorbance was recorded at 530 nm (A_530_) and 657 nm (A_657_). Here, A_530_ corresponds to the peak absorbance of Anth, and A_657_ was used for pheophytin correction. The expression A_530_ – (0.25×A_657_) was used to calculate the effective absorbance by Anth. A standard curve of cyanidin-3-*O*-glucoside (Merck KGaA, Darmstadt, Germany) was used for calculating Anth content per unit leaf FW.

### Color feature extraction and comparison with pigment contents

Digital color features of whole leaves were extracted using a customized image processing pipeline in Python program (www.python.org) by implementing *numpy* and *cv2* libraries. Within the pipeline, features from three color spaces, namely, RGB, HSV, and L*a*b*, were extracted for all pixels within the leaf boundary (min. 5000 pixels) for calculating the average value of each color feature for each sample. Linear and nonlinear correlation of all color features was performed for each type of pigment, and represented using scatter plots with best-fit trendlines and coefficient of determination (*R^2^*; 95% confidence interval). Subsequently, color space data were subjected to principal component analysis (PCA) with and without pigment contents to visualize variations across RLV, RGLV, and GLV categories in terms of color space profiles, i.e. RGB, HSV, and L*a*b* datasets, as well as for the combination of these three color spaces, henceforth referred to as All_3. For this, a customized PCA pipeline was designed in Python using *scikit-learn* libraries (www.scikit-learn.org) [35], with a threshold of >99% variance explained. Data were normalized prior to the analysis. PCA biplots were generated using the first two PCs (PC1, PC2) to visualize the results.

### Prediction of pigment contents by digital color features

Features from the different color spaces were used for predicting pigment contents following three different modeling approaches: (1) Partial Least Squares Regression (PLSR), (2) Support Vector Regression (SVR), and (3) Random Forest Regression (RFR). Herein, PLSR is a fundamental ML tool that can predict a single output variable using multiple input variables by creating latent variables or “components” which are linear combinations of the actual variables [36]. In contrast, SVR is a more advanced ML tool capable of creating linear and nonlinear equations in high-dimensional space [37], whereas RFR is a ML technique that implements a combination of “decision trees” depending on the values of randomly sampled vectors to generate a “random forest” for prediction [38]. All ML methods were tested using the RGB, HSV, L*a*b*, and All_3 color space datasets. Modeling was performed in Python using *scikit-learn* libraries. An overview of process parameters for modeling is provided in Table 1.

**Table 1.** Overview of machine learning parameters.

Parameter	Value	Details
Sample size	320	Training: 256
		Validation: 64
Model instances	25	No. of cross-validations: 5
		No. of random states: 5
Machine learning method	PLSR	No. of components = No. of color features
	SVR	Kernel: *Lin*, *Pol*, *Rbf*
		*C*: 0.1, 1, 10, 100
		*ε*: 0.01, 0.1, 1, 10
		*γ*: 0.001, 0.01, 0.1, 1 (*Pol* and *Rbf* only)
		Degree: 2, 3, 4, 5 (*Pol* only)
	RFR	Estimators (*n_estim*): 5, 10, 50
Color datasets	4	RGB, HSV, L*a*b*, All_3

Methods: PLSR, Partial Least Squares Regression; SVR, Support Vector Regression; RFR, Random Forest Regression. Kernels: *Lin*, *linear*; *Pol*, *polynomial*; *Rbf*, *radial basis function*. *C*, regularization parameter balancing model fit and complexity; *γ*, parameter setting the range of influence for a single training point; *ε*, margin of tolerance with no penalty for errors; Degree, degree of polynomial function. RGB: Red, Green, and Blue; HSV: Hue, Saturation, and Value; L*a*b*: Lightness, Redness-greenness, and Yellowness-blueness; All_3: RGB, HSV, and L*a*b* data combined.

Briefly, 25 instances for each type of model were generated using 5-fold cross-validation with five different random states, i.e. randomized shuffling of data prior to segregation of training and validation datasets. For PLSR, preliminary tests revealed that a higher number of model components improved prediction. Hence, PLSR models were created with the same number of components as the color features in each dataset, i.e. *n *=* *3 for RGB, HSV, and L*a*b*, and *n *=* *9 for All_3. The SVR models were tested for three kernels or mathematical relations, viz., *linear* (*Lin*), *polynomial* (*Pol*), and *radial basis function* (*Rbf*; Gaussian model). Additionally, training of SVR models was optimized by fine-tuning four additional hyperparameters as follows: (1) *C*, regularization parameter balancing model fit and complexity; (2) *γ*, setting the range of influence within the model for a single training point; (3) *ε*, margin of tolerance with no penalty for errors; (4) degree of polynomial function (Table 1). Similarly, performance of RFR models was evaluated for 5, 10, and 50 estimators (RFR_*5*, RFR_*10*, RFR_*50*). The threshold of RFR estimators was determined following preliminary tests using *n *=* *1, 5, 10, 50, 100, 250, 500, and 1000 estimators (*n_estim*), wherein *n_estim* > 50 resulted in only marginal improvement (<1%) although the data processing time increased considerably. Subsequently, relative importance (*RI*) of all color features for predicting each type of pigment content using the different ML methods was assessed in Python via the *permutation_importance* function of the *scikit-learn* package. Herein, five iterations for each type of model were generated with all color features by changing the random state, with ten repetitions of permutations for each iteration.

### Assessing the impact of Anth content on model output

Predictive performance of the PLSR, SVR, and RFR models for each pigment type was further analyzed by grouping the samples based on actual Anth content. For this, two instances of PLSR, SVR, and RFR models were evaluated by training (*n *=* *256 samples) and validation (*n *=* *64 samples) with nonidentical datasets using the best-performing colorimetric dataset (RGB, HSV, L*a*b*, or All_3) and optimized modeling parameters, i.e. hyperparameters for SVR and *n_estim* for RFR. The models were generated such that the validation datasets were mutually exclusive for both instances. Actual and predicted pigment contents were collated for both model instances, followed by grouping of samples based on actual Anth content as follows: high Anth (HA; Anth ≥ 0.5 mg/g FW); medium Anth (MA; 0.07 ≤ Anth < 0.5 mg/g FW); low Anth (LA; 0.01 ≤ Anth < 0.07 mg/g FW); and very low Anth (VLA; Anth < 0.01 mg/g FW). Subsequently, predictive accuracy across the Anth content-based categories was assessed by calculating mean absolute error (MAE) and mean absolute percentage error (MAPE) between the actual and predicted values.

### Statistical analysis

Overlap between the RLV, RGLV, and GLV samples for all colorimetric scatter plots as well as PCA biplots was quantified by calculating the scaled Euclidean distance between the centroids of each group (Δ*C*), where Δ*C *=* *0 indicates perfect overlap, and Δ*C *=* *1 indicates maximum separation. Goodness-of-fit for all prediction models was represented by *R^2^* and root-mean-squared error (RMSE) at a confidence interval of 95%.

## Results and discussion

### Comparison of pigment contents and digital color attributes

Since leaf color results from the interaction of incident visible light with the blend of pigments present, it can be considered a dynamic attribute of the leaf, which varies in response to physiological changes that affect pigment composition. Therefore, noninvasive estimation of leaf pigment content through digital color analysis requires a thorough evaluation of variations in leaf color profiles associated with different pigment blends. Simultaneous assessment of leafy vegetables with diverse pigment compositions in the current investigation allowed for a detailed exploration of this phenomenon.

In the present cohort, the contents of Chl and Car were similar across all six leafy vegetables, albeit with considerable range, i.e. 0.06–2.23 mg/g FW and 0.03–0.37 mg/g FW for Chl and Car, respectively (Table 2). Conversely, the range of Anth content varied markedly across the different types of leafy vegetables as expected. In particular, samples of RLV, i.e. PB and RPC, had the highest Anth contents amongst all, i.e. between 0.07 and 3.41 mg/g FW (Table 2). In comparison, RGLV samples (SK) had relatively lower Anth contents (<0.34 mg/g FW), whereas the GLV samples, i.e. GB, GPC, and WR, had the lowest overall Anth levels (<0.07 mg/g FW).

**Table 2.** Range of pigment contents (mg/g FW) in the leaf samples selected for the present study.

Plant	PB	RPC	SK	GB	GPC	WR
Chlorophyll	0.8–2.06	0.48–1.93	0.11–2.05	0.27–1.53	0.06–2.23	0.31–2.12
Carotenoid	0.15–0.36	0.09–0.32	0.1–0.37	0.07–0.25	0.03–0.29	0.12–0.35
Anthocyanin	0.44–3.41	0.07–1.02	0.001–0.33	0.001–0.02	0.001–0.06	0.001–0.01
Category	RLV	RLV	RGLV	GLV	GLV	GLV
*n*	60	40	100	40	40	40

Plants: GB, Greek basil; GPC, Green pak choi; PB, Purple basil; RPC, Red pak choi; SK, Scarlet kale; WR, Wasabi rocket. Categories based on the visual appearance: GLV, green leafy vegetable; RGLV, red-green leafy vegetable; RLV, red leafy vegetable. *n*, number of leaves used.

Plotting of these pigment contents with the digital color features revealed diverse trends for the different types of plants (Supplementary Figs. S1–S3). Notably, while samples of RGLV (*n *=* *100) and GLV (*n *=* *120) showed considerable overlap for most of the color features (0.02 < Δ*C* < 0.23; Supplementary Table S1), RLV samples (*n *=* *100) were plotted more distinctly from the other two groups in general (0.19 < Δ*C* < 0.67; Supplementary Table S1). This indicates that the relation between pigment contents and digital color features of GLV and RGLV were highly similar, whereas RLV presented a clearly divergent trend.

**Figure 2:**
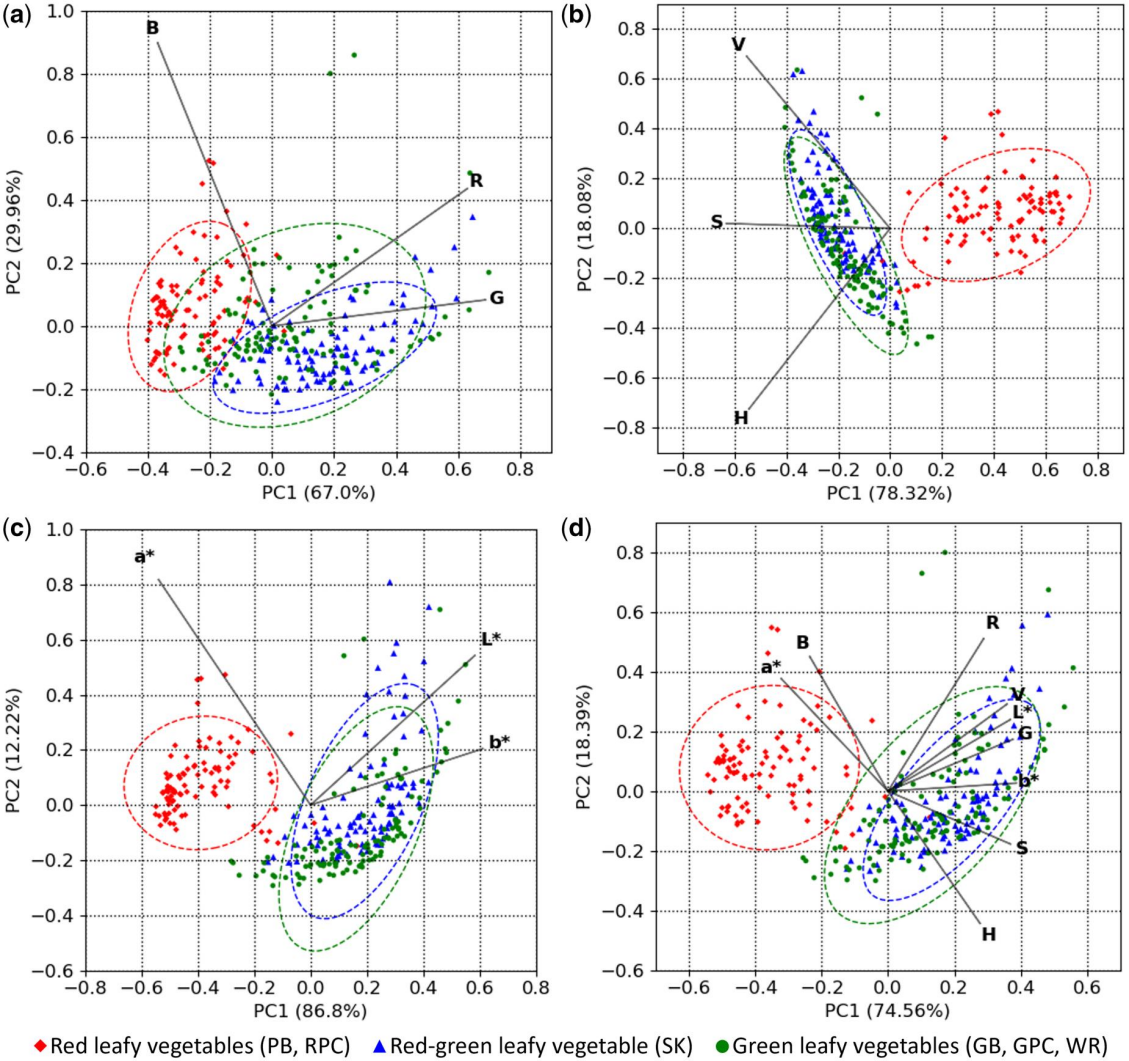
PCA biplots depicting the variations in digital color profiles of leafy vegetables with different visual profiles. Color space datasets: (a) Red, Green, Blue (R, G, B); (b) Hue, Saturation, Value (H, S, V); (c) Lightness, Redness–greenness, Yellowness–blueness (L*, a*, b*); (d) all three color spaces combined. PC1 and PC2, first and second principal components, with values in parentheses indicating variance explained by the respective PC. Ellipses represent 95% confidence intervals. Plants within different categories: Purple basil (PB), Red pak choi (RPC), Scarlet kale (SK), Greek basil (GB), Green pak choi (GPC), and Wasabi rocket (WR). Sample sizes: Green leafy vegetables, *n* = 120; Red-green leafy vegetable, *n* = 100; Red leafy vegetables, *n* = 100

The tendency is represented more concisely by the PCA biplots obtained upon analyzing the four colorimetric datasets, i.e. RGB, HSV, L*a*b*, and All_3 (Fig. 2). Herein, the strong overlap between RGLV and GLV samples (0.05 < Δ*C* < 0.15; Supplementary Table S1), with the RLV samples forming a distinct cluster in most cases (0.29 < Δ*C* < 0.56; Supplementary Table S1), reiterates the impact of Anth on leaf digital color profile. The observation was contrary to our expectation of RGLV samples being clustered between RLV and GLV due to intermediate Anth contents. This suggests that the Anth content of RGLV samples was likely not high enough to overcome Chl-dominance and elicit a distinctive shift in the colorimetric profile with respect to GLV.

While the PCA biplots with HSV, L*a*b*, and All_3 datasets showed negligible overlap between RLV and GLV samples (Fig. 2b–d; Δ*C* > 0.45), the biplot with RGB data showed partial overlap between these two groups (Fig. 2a; Δ*C *=* *0.315). Hence, it could be inferred that RGB data did not account for the variations in Anth content as strongly as HSV and L*a*b* color spaces, possibly due to the segregation of redness and greenness into two channels of the RGB color space, with the redness-greenness transition in leaf color being a characteristic indicator of changing Anth status. The subsequent PCA of color features with pigment contents (Supplementary Fig. S4) further revealed that RLV samples with very low Anth contents, as indicated by their position away from the Anth vector, overlapped with the GLV and RGLV groups. This suggests that, like the RGLV samples, RLV samples with Anth content below a certain threshold had colorimetric profiles highly similar to GLV samples, dictated predominantly by the Chl content.

Nonetheless, the observations highlight the impact of Anth on leaf digital color profiles, clearly demonstrating the misleading or “red herring” shift in colorimetric features caused by high Anth contents. Such deviations pose a challenge in implementing simplistic broad-spectrum digital color analysis models for estimating pigment contents in green- and red-leaved crop species simultaneously due to limited generalizability, necessitating the application of advanced approaches such as ML. The subsequent sections delve deeper into the possibilities and limitations of generalized ML-based models for pigment content estimation using digital color data as revealed by our analyses.

### Predicting Chl content

Owing to the importance of Chl as a key indicator of plant health status and nutritional value, estimating the content of this pigment has been of interest for crop scientists and cultivators since many decades. While the process conventionally relied mainly on spectrophotometric estimations using leaf extracts as proposed by pioneering studies [33, 39], introduction of Chl meters such as SPAD [40] was a major advancement as it enabled nondestructive estimations for the first time. Further, with concomitant improvements in digital imaging as well as data processing technologies in the past two decades, a large number of studies have demonstrated the application of various ML-based approaches such as SVR, RFR, back-propagation neural network, multilayer perceptron, ridge regression, and gradient boosting decision tree for high-throughput prediction of Chl content via RGB and multispectral imaging [17, 19, 20, 41–45]. Since all such studies have presented the findings pertaining to single crops, the next step in advancing Chl estimations would be the development of generalized models that could be applied to multiple crops simultaneously, including both green-leaved and anthocyanic varieties, as presented herein.

**Figure 3:**
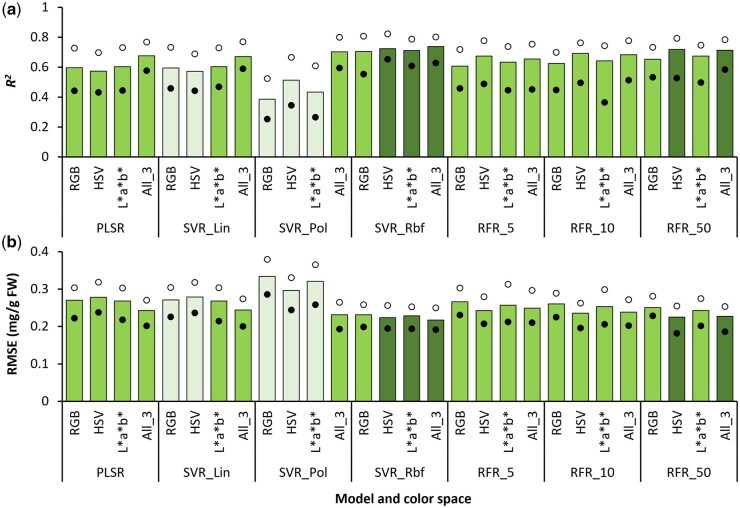
Accuracy of predicting chlorophyll content via different combinations of modeling approaches and color space datasets as indicated by the coefficient of determination (*R^2^*; a) and root-mean-squared error (RMSE; b). Vertical bars indicate mean values, whereas the solid and open circles represent the lower and upper ranges, respectively, for *n* = 25 instances of each type of model generated via 5-fold cross-validation with five different random states. Modeling methods: PLSR, Partial Least Squares Regression; SVR, Support Vector Regression with *linear* (*Lin*), *polynomial* (*Pol*), and *radial basis function* (*Rbf*) kernels; RFR_*n*, Random Forest Regression with *n* estimators. Color spaces: RGB, Red, Green, Blue; HSV, Hue, Saturation, Value; L*a*b*, Lightness, Redness–greenness, Yellowness–blueness; All_3, combined dataset of RGB, HSV, and L*a*b* color spaces. Darker and lighter shades on bars indicate the best and poorest outcomes, respectively

In general, accuracy of predicting Chl content differed markedly for the different ML methods and color space datasets tested (Fig. 3). Amongst all approaches, estimation of Chl content was most accurate when SVR_*Rbf* models were trained using the All_3 dataset (*R^2^* = 0.738, RMSE = 0.217 mg/g FW). Further, implementing the same ML method with individual color spaces resulted in slightly less accurate Chl content estimates (0.7 < *R^2^* < 0.725, 0.22 < RMSE < 0.24 mg/g FW). In contrast, using SVR_*Pol* yielded considerably inaccurate Chl predictions with individual color space datasets (0.38 < *R^2^* < 0.52, 0.29 < RMSE < 0.34 mg/g FW) as compared to the All_3 dataset (*R^2^* = 0.704, RMSE = 0.232 mg/g FW). Although a similar trend was also observed for the linear models, viz., PLSR and SVR_*Lin*, the difference in accuracy was relatively lesser between the models created using individual color spaces (0.57 < *R^2^* < 0.61, 0.268 < RMSE < 0.278 mg/g FW) and the All_3 dataset (*R^2^* ∼ 0.67, RMSE ∼ 0.24 mg/g FW).

In contrast to the PLSR and SVR models, where the All_3 dataset gave the best results, accuracy of predictions using RFR_*50* (Fig. 3) was marginally better with HSV values (*R^2^* = 0.719, RMSE = 0.225 mg/g FW) as compared to the All_3 dataset (*R^2^* = 0.713, RMSE = 0.228 mg/g FW). Overall, RFR models trained using HSV and All_3 datasets had better predictions compared to RGB and L*a*b* dataset-based models for all three *n_estim* levels. Further, increasing *n_estim* improved the overall accuracy progressively for all colorimetric datasets, and only the SVR_*Rbf* models outperformed the RFR_*50* models.

Interestingly, although R was identified as one of the most important features for Chl estimation (0.23 < *RI* < 0.71; Supplementary Table S2), which is understandable considering the strong correlation of R with Chl content (*R^2^* = 0.715, *n *=* *320; Supplementary Fig. S1a), predictions using RGB data alone did not yield very high accuracies for any of the ML methods. This suggests that instead of relying on information provided by individual color features, the prediction models took into consideration the underlying relations of all available color features. Consequently, a synergistic effect of combining data from multiple color spaces for better Chl content prediction was observed for all three modeling approaches.

Comparing the best and worst outcomes of *n *=* *25 instances for each type of model indicated that predictions were most consistent with SVR_*Rbf* modeling using HSV and All_3 datasets (Fig. 3), i.e. variation of outcomes across model instances was the least (Δ*R^2^* < 0.174, ΔRMSE < 0.062 mg/g FW). In contrast, outcomes were most inconsistent when L*a*b* was used to train SVR_*Pol* and RFR_*10* models (Δ*R^2^* > 0.34, ΔRMSE > 0.09 mg/g FW). Hence, selection of the *Rbf* kernel was beneficial for SVR-based models during Chl estimation, whereas increasing the number of estimators led to lower variability in the RFR models. Additional trials with more diverse plant varieties and ML methods would allow further optimization of accuracy and consistency.

### Predicting Car content

**Figure 4:**
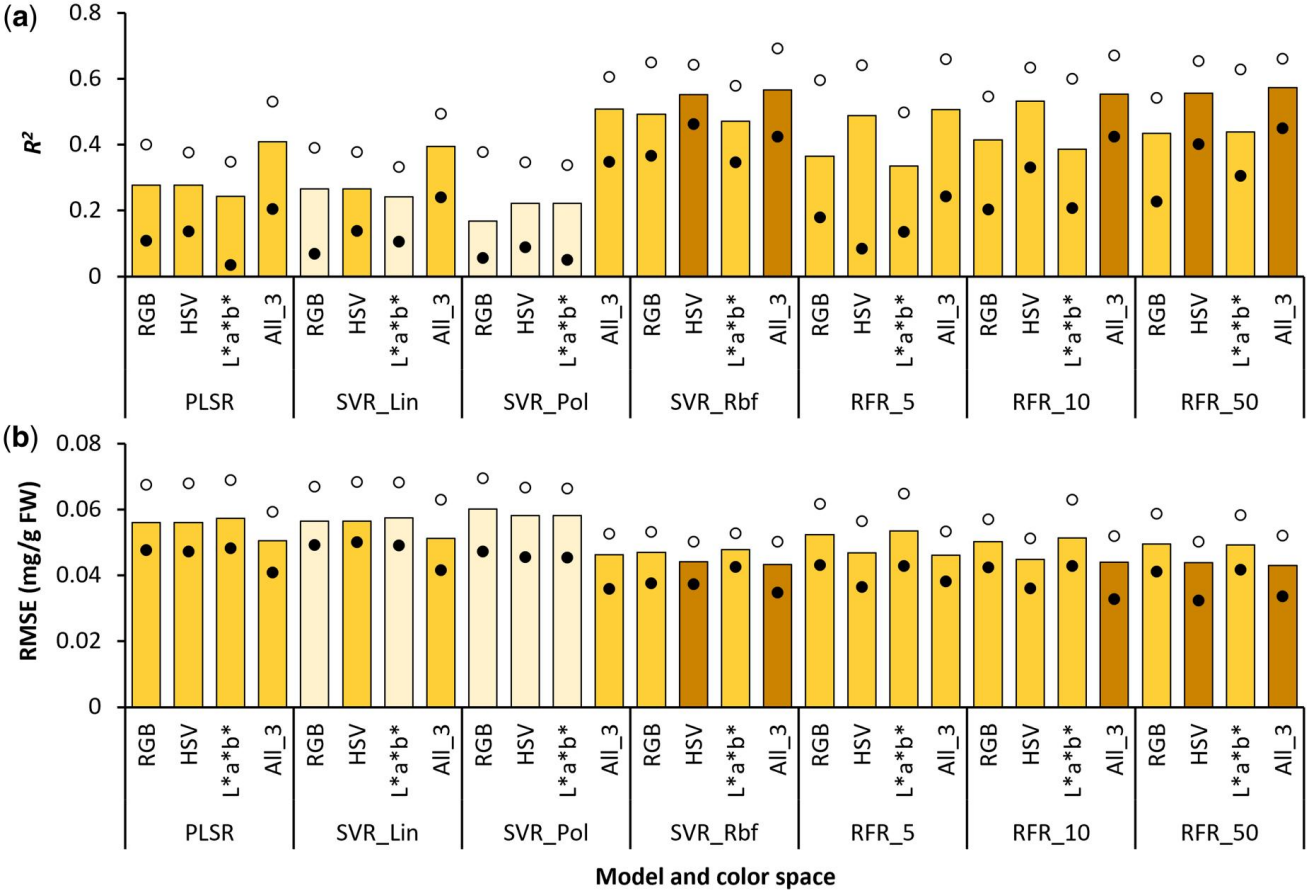
Accuracy of predicting carotenoid content via different combinations of modeling approaches and color space datasets as indicated by the coefficient of determination (*R^2^*; a) and root-mean-squared error (RMSE; b). Vertical bars indicate mean values, whereas the solid and open circles represent the lower and upper ranges, respectively, for *n* = 25 instances of each type of model generated via 5-fold cross-validation with five different random states. Modeling methods: PLSR, Partial Least Squares Regression; SVR, Support Vector Regression with *linear* (*Lin*), *polynomial* (*Pol*), and *radial basis function* (*Rbf*) kernels; RFR_ *n*, Random Forest Regression with *n* estimators. Color spaces: RGB, Red, Green, Blue; HSV, Hue, Saturation, Value; L*a*b*, Lightness, Redness–greenness, Yellowness–blueness; All_3, combined dataset of RGB, HSV, and L*a*b* color spaces. Darker and lighter shades on bars indicate the best and poorest outcomes, respectively

Similar to the Chl estimation models, prediction of Car content was most accurate when the All_3 dataset was used with different modeling algorithms (Fig. 4). In particular, RFR_*50* (*R^2^* = 0.573, RMSE = 0.043 mg/g FW) and SVR_*Rbf* (*R^2^* = 0.566, RMSE = 0.0433 mg/g FW) were the two most accurate modeling approaches when the All_3 dataset was used. HSV color data also gave relatively good results (*R^2^* > 0.55, RMSE < 0.045 mg/g FW) with SVR_*Rbf* and RFR_*50* models. However, comparing the average variation in *R^2^* and RMSE values for the different color datasets indicated that the outcomes were more consistent across *n *=* *25 model instances for the SVR_*Rbf* models (Δ*R^2^* = 0.24, ΔRMSE = 0.0135 mg/g FW) as compared to RFR_*50* models (Δ*R^2^* = 0.275, ΔRMSE = 0.0176 mg/g FW). In contrast, the least reliable results were obtained when data from the three color spaces were used independently with SVR_*Pol* (0.169 < *R^2^* < 0.222, 0.058 < RMSE < 0.061 mg/g FW), followed by PLSR and SVR_*Lin* (0.241 < *R^2^* < 0.278, 0.056 < RMSE < 0.058 mg/g FW). It can thus be inferred that, like Chl content, prediction of Car content was more accurate when appropriate SVR and RFR parameters were applied, particularly with All_3 and HSV datasets.

In the past, Car estimation has been extensively dependent on leaf extract-based measurements using spectrophotometry and high-performance liquid chromatography [33, 46, 47]. Since the spectral attributes of Car overlap strongly with the more dominant Chl in green leaves, as well as with both Chl and Anth in anthocyanic leaves [47–49], previous studies have even utilized techniques such as reflectance spectroscopy, mass spectrometry, and hyperspectral imaging to dissect leaf spectral traits for estimating Car content noninvasively [50–54]. However, the intricacies of data analysis presented therein limit direct application of these technologies to commercial farming operations with diverse plants.

To simplify the process, digital imaging-based studies on green-leaved plants demonstrated the strong inverse relationship of Car with the G channel of the RGB color space [27, 28]. This relationship is attributed to the absorptive capacity of Car in the green waveband [46, 55]. Consequently, G values could have been useful for predicting Car content. However, because Anth molecules also have a strong absorbance in the green waveband [49], good correlation between Car content and G values was not observed in our analyses (*R^2^* = 0.196, *n *=* *320), especially due to an abrupt shift in the G values of Anth-rich plants (Supplementary Fig. S2b). Further, G was deemed as an important feature only for the PLSR model (*RI *=* *0.485; Supplementary Table S2). Hence, the present findings highlight the likelihood of interference by Anth on Car estimation in Anth-rich plants using G values.

As anticipated, the overall accuracy of predicting Car content (0.16 < *R^2^* < 0.58; Fig. 4) was not very high. This relatively low accuracy of Car content prediction was primarily due to “pigment masking,” a phenomenon wherein the contribution of the target pigment to leaf color is overshadowed by the presence of high concentrations of other pigments with overlapping absorbance spectra [56–58]. Since most of the samples used in this study had very high contents of Chl, along with high Anth in RLV samples, it is possible that the impact of Car on the digitally recorded color features was not discernible due to masking by the other two pigments. Consequently, only approximate Car estimates (*R^2^* < 0.6) could be obtained using the digital images.

It is worth mentioning here that while high Chl contents can completely mask Car, concentration of Chl and Car within a leaf is strongly correlated, as reported in various plant species [28, 59–62]. This trend was also observed in our study upon comparing Chl and Car contents of leaf samples for each of the six leafy vegetables individually (0.68 < *R^2^* < 0.93; Supplementary Table S3). Furthermore, preliminary analyses with the current samples yielded more accurate indirect Car content estimates (*R^2^* = 0.69–0.79, RMSE = 0.031–0.038 mg/g FW; data not shown) using predicted Chl content values and plant-specific Chl vs. Car correlation equations (Supplementary Table S3) as compared to direct Car content estimates using colorimetric data and ML (Fig. 4). Hence, from the perspective of feasibility and considering the possibility of Car masking by other pigments, indirect estimation of Car using predicted Chl content could be deemed more reliable than attempting direct digital color-based Car estimation, especially for Anth-rich varieties. However, further testing of species- and variety-specific models would be needed to use this method with higher fidelity, especially because the Chl: Car balance may differ significantly across plant genotypes.

### Predicting Anth content

Estimation of leaf Anth, like Car, has predominantly relied on leaf extract-based measurements [63–65]. While various studies have demonstrated the potential of assessing Anth content nondestructively using methods such as hyperspectral imaging and reflectance spectroscopy [51, 66–68], handheld devices such as ACM-200 have also been developed to streamline the process [69]. Although such approaches presented the possibility of nondestructive Anth estimation, bottlenecks such as dependence on specialized high-end instrumentation in the former and labor intensiveness for the latter remained.

Nonetheless, a few recent studies have demonstrated the possibility of estimating Anth content using digital imaging. For instance, Askey *et al*. [70] carried out experiments using green as well as Anth-rich *Arabidopsis* genotypes comparing different ML-based regression models to assess Anth accumulation through digital imaging as a means for evaluating plant stress. In a later investigation by Kim and van Iersel [30], two red-leafed lettuce cultivars were used for quantifying Anth via the Normalized Difference Anth Index, calculated as [I_R_ − I_G_]/[I_R_ + I_G_], wherein I_R_ and I_G_ indicate pixel intensity in the red and green wavebands, respectively. Similarly, Clemente *et al*. [31] reported Anth estimation in lettuce by employing the Green Leaf Index, i.e. [2G − R − B]/[2G + R + B]. The present observations augment these findings by contributing to the development of more comprehensive Anth prediction models that could be implemented for assessing the nutritional value of a diverse range of leafy vegetables simultaneously.

**Figure 5:**
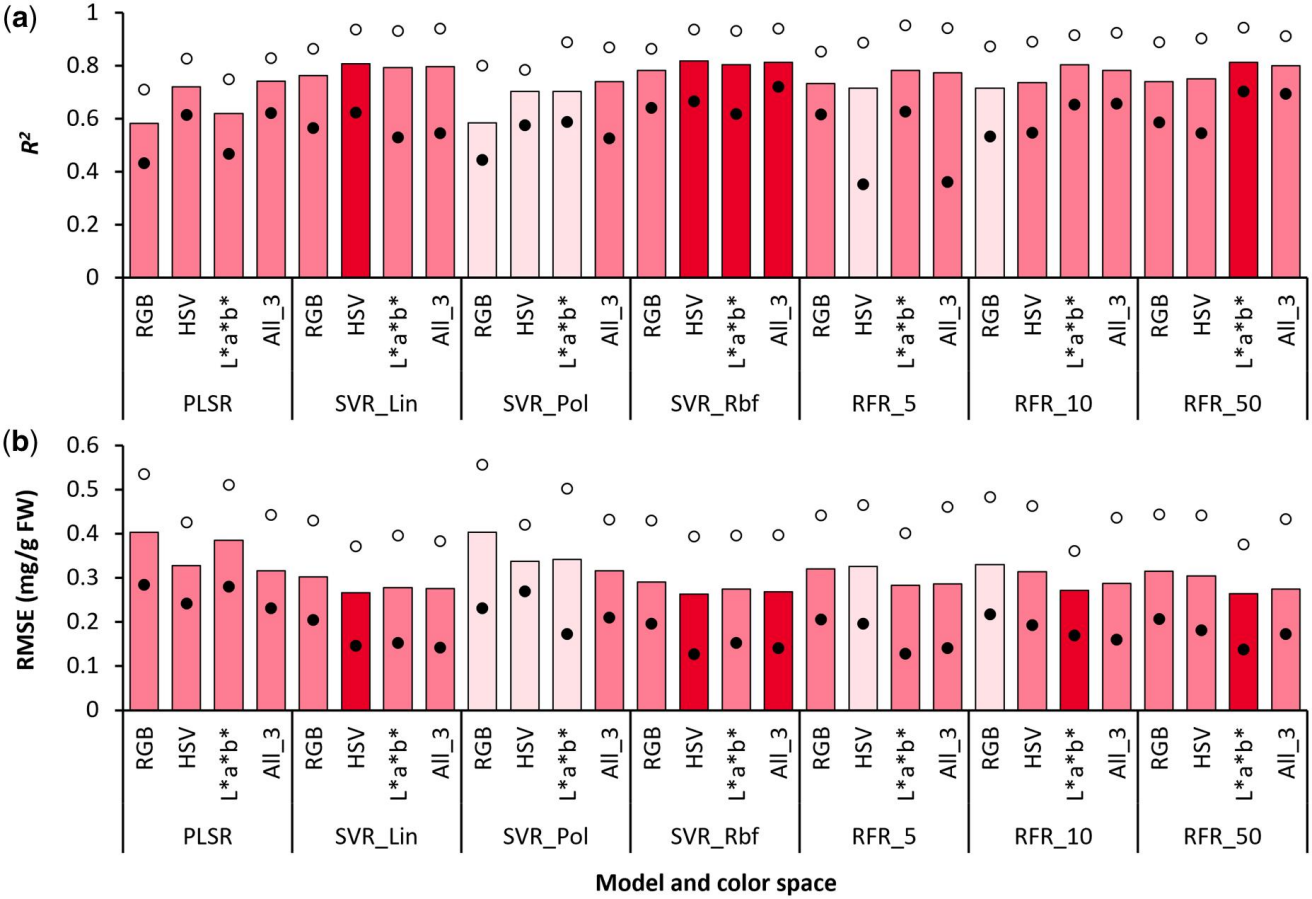
Accuracy of predicting anthocyanin content via different combinations of modeling approaches and color space datasets as indicated by the coefficient of determination (*R^2^*; a) and root-mean-squared error (RMSE; b). Vertical bars indicate mean values, whereas the solid and open circles represent the lower and upper ranges, respectively, for *n* = 25 instances of each type of model generated via 5-fold cross-validation with five different random states. Modeling methods: PLSR, Partial Least Squares Regression; SVR, Support Vector Regression with *linear* (*Lin*), *polynomial* (*Pol*), and *radial basis function* (*Rbf*) kernels; RFR_*n*, Random Forest Regression with *n* estimators. Color spaces: RGB, Red, Green, Blue; HSV, Hue, Saturation, Value; L*a*b*, Lightness, Redness–greenness, Yellowness–blueness; All_3, combined dataset of RGB, HSV, and L*a*b* color spaces. Darker and lighter shades on bars indicate the best and poorest outcomes, respectively

In our study, SVR_*Rbf* models yielded the best results for Anth prediction across different color datasets (0.78 < *R^2^* < 0.82, 0.26 < RMSE < 0.29 mg/g FW; Fig. 5). In contrast, PLSR and SVR_*Pol* models using RGB data produced the least accurate predictions (*R^2^* ∼ 0.58, RMSE ∼ 0.4 mg/g FW), followed by PLSR models implementing L*a*b* data (*R^2^* = 0.62, RMSE = 0.38 mg/g FW). All other PLSR and SVR models gave relatively reliable predictions (*R^2^* > 0.7, RMSE < 0.34 mg/g FW) irrespective of the color dataset used (Fig. 5). Similarly, all RFR models also provided good estimates of Anth content (*R^2^* > 0.7, RMSE < 0.33 mg/g FW), and increasing *n_estim* from 5 to 50 increased the accuracy by a small margin (*n_estim *=* *5: 0.714 < *R^2^* < 0.782, 0.282 < RMSE < 0.326 mg/g FW; *n_estim *=* *50: 0.74 < *R^2^* < 0.813, 0.264 < RMSE < 0.315 mg/g FW; Fig. 5).

For the PLSR- and SVR-based models, both HSV and All_3 datasets gave highly accurate results (0.702 < *R^2^* < 0.818, 0.262 < RMSE < 0.338 mg/g FW; Fig. 5), followed by models utilizing the L*a*b* dataset (0.618 < *R^2^* < 0.805, 0.274 < RMSE < 0.385 mg/g FW). However, the RFR algorithm performed most reliably when trained with L*a*b* data (0.782 < *R^2^* < 0.814, 0.264 < RMSE < 0.283 mg/g FW), outperforming RFR models trained using the All_3 dataset (0.773 < *R^2^* < 0.799, 0.274 < RMSE < 0.287 mg/g FW), while the HSV-based RFR models lagged behind by a clear margin (0.714 < *R^2^* < 0.751, 0.304 < RMSE < 0.326 mg/g FW). This is in contrast to the results of Chl estimation (Fig. 3), wherein HSV-based RFR models performed better than the L*a*b*-based RFR models. This observation highlights the importance of compatibility between the colorimetric dataset and the modeling algorithm for accurately estimating specific pigment types.

Interestingly, the relatively lower accuracy of RGB-based Anth prediction models (Fig. 5) highlights the limitations of this color space in accurately capturing the transitions between leaf greenness and redness as Anth content increases, as discussed in the section on “Comparison of pigment contents and digital color attributes”. In contrast, correlation analyses (Supplementary Fig. S3) and evaluation of importance (Supplementary Table S2) revealed that HSV and L*a*b*-based features, namely, H and a*, reflected the change in Anth content strongly (*R^2^* > 0.78, *n *=* *320), and were deemed to be the most important features for Anth prediction with all nonlinear algorithms (0.19 < *RI* < 0.66). Notably, both these features account for the transition between redness and greenness across a continuous scale, a characteristic visual change observed in leaf color due to variations in Anth content. Hence, considering these factors along with the better performance of HSV and L*a*b*-based models, both these colorimetric datasets could be chosen for Anth predictions, but with due consideration to consistency of predictions for the selected algorithm–dataset combination.

As observed, consistency of Anth prediction as per the difference between highest and lowest values of both *R^2^* and RMSE observed across *n *=* *25 model instances was highest for the PLSR models utilizing HSV and All_3 datasets, as well as for the SVR_*Pol* models generated using HSV data (Δ*R^2^* < 0.214, ΔRMSE < 0.212 mg/g FW). Interestingly, models with better overall Anth prediction accuracy, such as SVR_*Rbf* and RFR_*50*, had higher variability between best and worst *R^2^* and RMSE values observed (Δ*R^2^* > 0.24, ΔRMSE > 0.23 mg/g FW). Hence, selection of Anth estimation model would require more careful consideration of consistency of outcomes along with prediction accuracy during practical implementation. Further tests with larger and more diverse training sample sizes and more complex ML methods would likely help optimize both factors.

### Impact of Anth content on pigment content prediction

**Figure 6:**
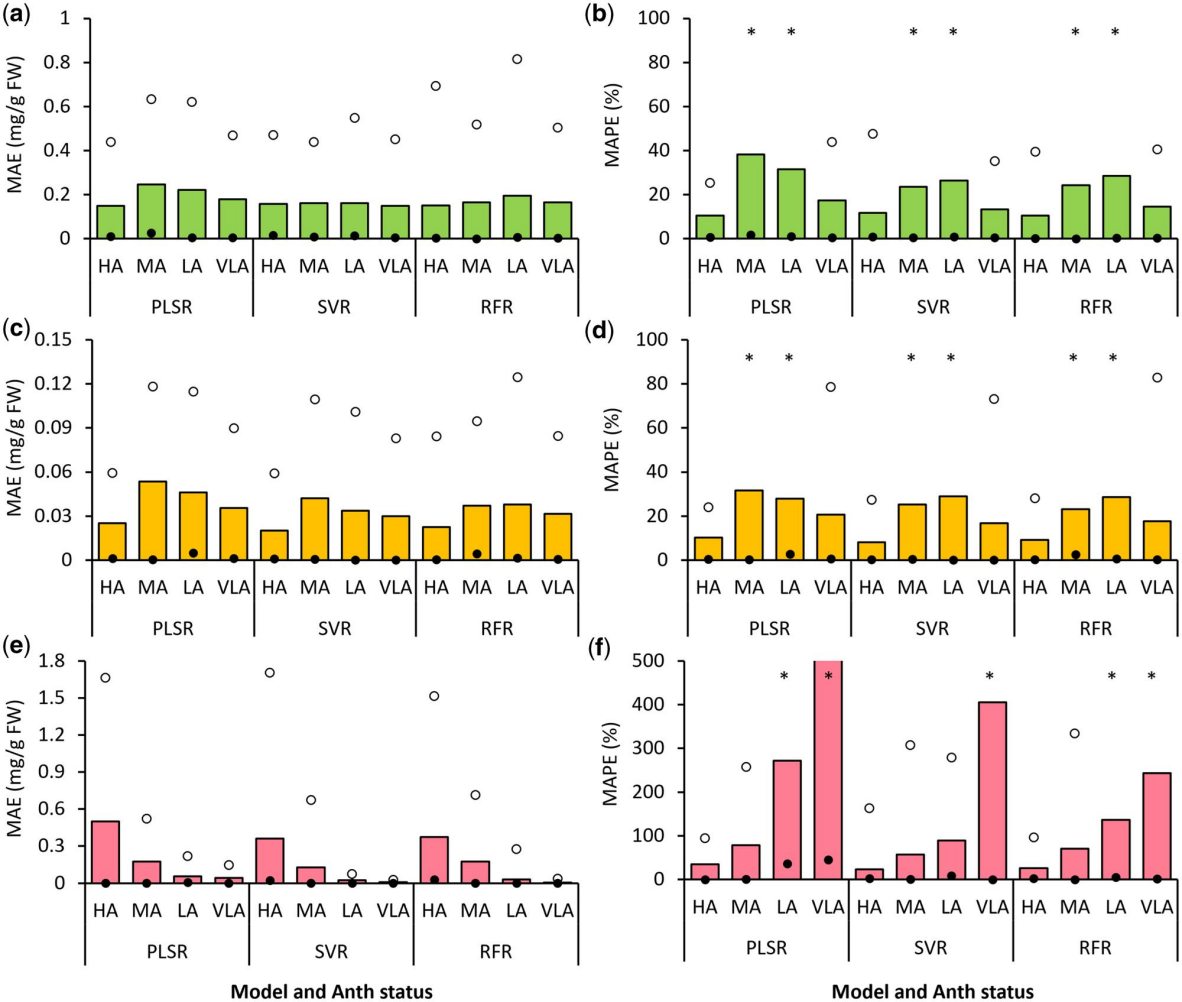
Mean absolute error (MAE) and mean absolute percentage error (MAPE) of predicting chlorophyll (a, b), carotenoid (c, d), and anthocyanin (Anth; e, f) contents via best-performing Partial Least Squares Regression (PLSR), Support Vector Regression (SVR), and Random Forest Regression (RFR) models (Table 3) for samples grouped as per observed leaf Anth content. Sample groups: HA, high Anth (Anth ≥ 0.5 mg/g FW, *n* = 28); MA, medium Anth (0.07 ≤ Anth < 0.5 mg/g FW, *n* = 25); LA, low Anth (0.01 ≤ Anth < 0.07 mg/g FW, *n* = 25); VLA, very low Anth (Anth < 0.01 mg/g FW, *n* = 50). Solid and open circles represent the lower and upper ranges, respectively. Values represent the combined output of two model instances, each created by using non-identical datasets for training (*n* = 256) and validation (*n* = 64). *MAPE upper limit values beyond the plotted axis range

While the above results (Figs. 3–5) depict the outcomes of pigment content prediction models for all six types of leafy vegetables combined, the present section compares the prediction outcomes for samples divided into four groups based on actual Anth content, i.e. HA, MA, LA, and VLA. For this the results from two instances of the best performing PLSR, SVR, and RFR models have been collated and evaluated in terms of MAE and MAPE (Fig. 6). An overview of model parameters considered for this assessment has been provided in Table 3.

**Table 3.** Best performing models for estimating each type of pigment content.

Pigment	Model	Parameters
Chlorophyll	PLSR	Dataset: All_3
	SVR	Dataset: All_3; Kernel: Rbf; *C*: 100; *ε*: 0.1; *γ*: 0.1
	RFR	Dataset: HSV; *n_estim *=* *50
Carotenoid	PLSR	Dataset: All_3
	SVR	Dataset: All_3; Kernel: Rbf; *C*: 10; *ε*: 0.01; *γ*: 0.1
	RFR	Dataset: All_3; *n_estim *=* *50
Anthocyanin	PLSR	Dataset: All_3
	SVR	Dataset: HSV; Kernel: Rbf; *C*: 10; *ε*: 0.01; *γ*: 1
	RFR	Dataset: L*a*b*; *n_estim *=* *50

Models: PLSR, Partial Least Squares Regression; SVR, Support Vector Regression; RFR, Random Forest Regression. Datasets: HSV, Hue, Saturation, Value; L*a*b*, Lightness, Redness-greenness, Yellowness-blueness; All_3, combined dataset of RGB (Red, Green, Blue), HSV, and L*a*b* color spaces. Hyperparameters: *Rbf*, *radial basis function* kernel; *C*, regularization parameter balancing model fit and complexity; *γ*, parameter setting the range of influence for a single training point; *ε*, margin of tolerance with no penalty for errors; *n_estim, n* estimators for RFR.

Here, comparison of MAE values for Chl prediction indicated that the SVR models did not differentiate between the VLA, LA, MA, and HA categories (Fig. 6a), although the mean MAPE values were distinctly higher for LA (MAPE = 26.3%) and MA (MAPE = 23.6%) groups compared to HA and VLA (MAPE < 13.5%; Fig. 6b). While the MAPE values for RFR and PLSR models were also higher for MA and LA samples (MAPE > 24.2%) compared to HA and LA (MAPE < 17.4%), higher MAE values were only observed for the LA group using the RFR models (MAE = 0.19 mg/g FW) as well as for both MA and LA with the PLSR models (MAE > 0.22 mg/g FW) compared to the other groups (MAE < 0.18 mg/g FW). Thus, it may be inferred that Chl predictions were more consistent for samples with very high and very low Anth contents, whereas intermediate Anth contents possibly confused the algorithms.

Likewise, Car content predictions were most accurate for the HA samples (MAE < 0.026 mg/g FW, MAPE < 10.3%; Fig. 6c and d) followed by the VLA group (MAE < 0.036 mg/g FW, MAPE < 20.1%) for all three types of models, in contrast to the MA and LA samples (0.035 < MAE < 0.054 mg/g FW, 23% < MAPE < 31.6%). Herein, while MAPE values were comparable across the different types of models, MAE values were generally higher for the PLSR-based models. The observations reiterate the possibility of model confusion for samples with intermediate Anth contents while predicting the concentration of other pigments, concurrently highlighting the better performance of methods such as SVR and RFR as compared to PLSR. Further investigations using larger training and test datasets with more varieties of plants having diverse pigmentations would provide a better insight into these aspects from the perspective of comparing outcomes for HA, MA, LA, and VLA samples.

Unlike Chl and Car predictions, MAE and MAPE showed a clear trend with increasing Anth content for Anth estimation models (Fig. 6e and f). In particular, MAE values showed a distinct increment with increasing Anth content, i.e. from 0.008–0.044 mg/g FW for VLA samples to 0.36–0.51 mg/g FW for HA samples. Conversely, the MAPE showed a reversed trend of increasing steadily with decreasing Anth content, i.e. from <35% for HA samples to >200% for VLA samples. This reversal in trend between MAE and MAPE suggests that while the absolute error of prediction was low for the LA and VLA samples, the magnitude of errors was too high compared to the actual Anth content of those samples. The plots of color features with Anth content (Supplementary Fig. S3) indicate that there was no discernible change in color features at very low Anth ranges. Hence, such minor variations in Anth content could not be reliably mapped onto variations in digital color feature values. Hence, the presently tested approach of image-based Anth estimation would be more practical for leaf samples with medium to reasonably high Anth contents. This inference takes into consideration the dominance of Chl on leaf color profile, which results in the masking of Anth in green-leaved samples, similar to Car masking observed across all sample categories. However, unlike the current approach wherein samples with very high to very low Anth contents were used simultaneously, prediction models created using samples with only medium to low Anth contents could be tested for higher sensitivity at lower Anth ranges.

In general, these observations highlight the “red herring” effect of varying Anth content on the performance of colorimetric data-based ML models for pigment content prediction. While numerous studies have proposed diverse protocols for nondestructive estimation of pigment contents via digital image analysis, none of the studies have addressed the possibility of generalizability across plant species or between anthocyanic and non-anthocyanic varieties in depth. Hence, our findings provide the first insight into the scope of developing holistic prediction models that take these aspects into account by proposing the use of broad-spectrum models that may be used for multiple crop species including green- as well as red-leaved varieties. Additional studies with bigger datasets derived from a more diverse cohort of green-leaved and Anth-rich plant varieties, and implementation of more advanced data processing tools such as deep learning, could enable further optimization of colorimetric data-based pigment content estimation.

## Conclusion

Our findings indicate that SVR_*Rbf* and RFR_*50* algorithms were most effective for predicting Chl, Car, and Anth contents following the generalized modeling approach due to their ability to account for more complex interrelations between multiple digital color features and pigment contents. Additionally, while combining the data from RGB, HSV, and L*a*b* color spaces was most effective for predicting pigment contents using the different algorithms, use of HSV and L*a*b* independently also provided reliable results when used with specific modeling parameters. Further, Chl and Anth could be estimated based on digital color features with high fidelity. Although similar estimations of Car content were not as accurate due to its masking by other pigments, the potential for more precise indirect estimations using predicted Chl content values remains to be fully explored. Furthermore, as the currently tested models were developed using a broad range of Anth contents, their sensitivity differed with Anth levels, highlighting the potential for testing smaller ranges of Anth for improving model precision for low Anth contents. Hence, while providing novel insights into the development of holistic ML-based models that may be implemented for estimating pigment contents across multiple green and red leafy vegetable species, our study also opens the avenue for further research in this direction by highlighting the limitations and future perspectives. Streamlining and implementation of such models in commercial practice would be greatly beneficial in real-time pre- and post-harvest monitoring of the nutritional quality of leafy vegetables.

## Supplementary Material

bpaf027_Supplementary_Data

## Data Availability

Data are available from the corresponding authors upon reasonable request.
