# Supplementary material for: Assessing nutritional pigment content of green and red leafy vegetables by image analysis: Catching the “red herring” of plant digital color processing via machine learning
Source: Biol Methods Protoc. 2025 Apr 9;10(1):bpaf027. doi: 10.1093/biomethods/bpaf027 (PMC12057810; doi:10.1093/biomethods/bpaf027)
Supplement: bpaf027_Supplementary_Data [file bpaf027_supplementary_data.pdf]

Assessing nutritional pigment content of green and red leafy vegetables by image analysis: Catching the "red herring" of plant digital color processing via machine learning

Avinash Agarwal[1,4]*, Filipe de Jesus Colwell[2], Viviana Andrea Correa Galvis[2], Tom R. Hill[3], Neil Boonham[1], Ankush Prashar[1]*

[1]School of Natural and Environmental Sciences, Newcastle University, Newcastle upon Tyne, UK
[2]Crop Science R&D Division, Infarm - Indoor Urban Farming B.V., Amsterdam, The Netherlands
[3]Faculty of Medical Sciences, Newcastle University, Newcastle upon Tyne, UK
[4]Institute for Bio- and Geosciences: Plant Sciences (IBG-2), Forschungszentrum Jülich GmbH, Jülich, Germany

***Correspondence:**
Ankush Prashar (ankush.prashar@newcastle.ac.uk)
Avinash Agarwal (avinash.agarwal.1612@gmail.com; a.agarwal@fz-juelich.de)

Supplementary material

Supplementary figure

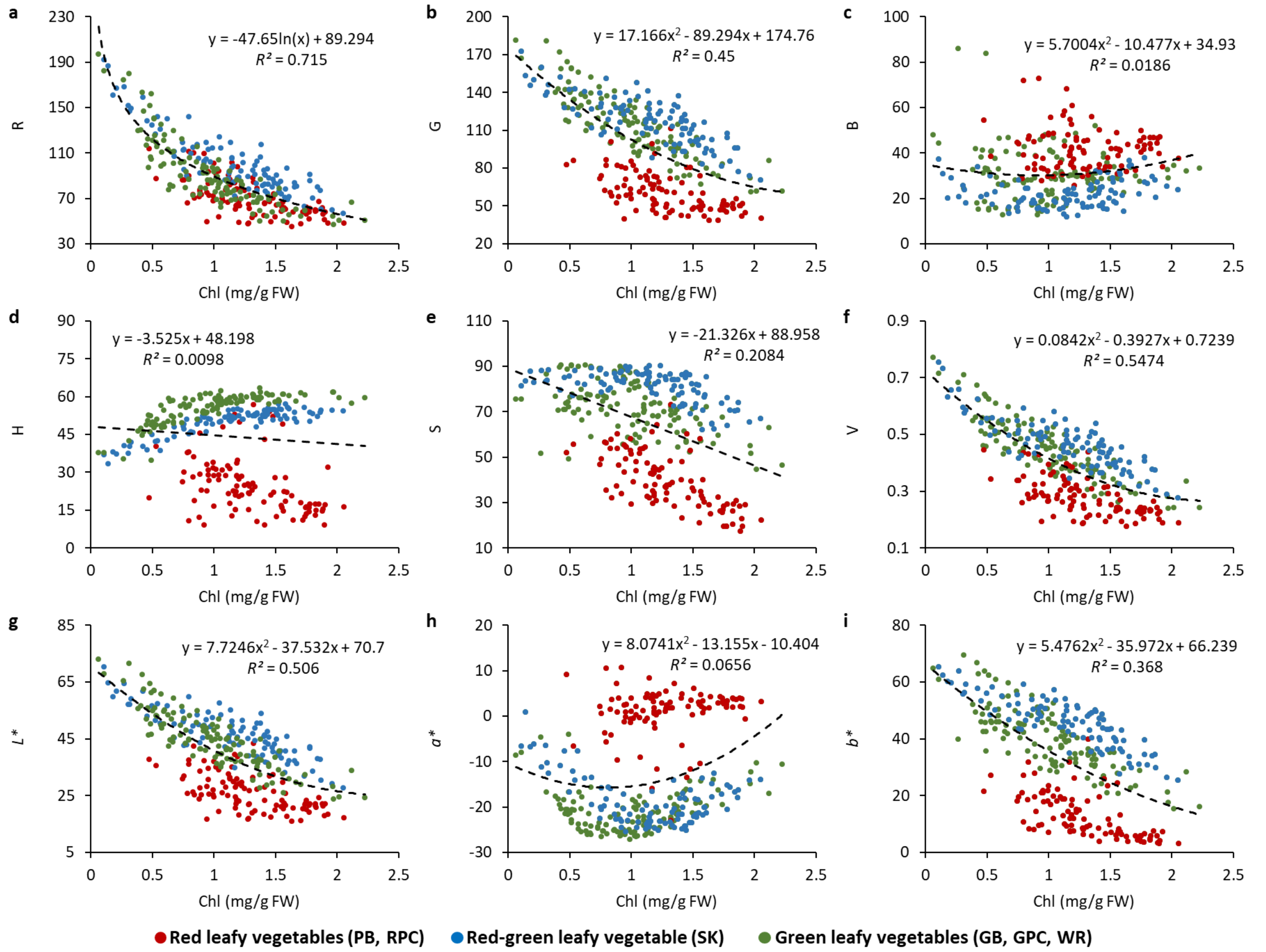

Fig. S1 Plots of leaf chlorophyll (Chl) content with different digital color features, i.e., Red, Green, Blue (RGB; a–c), Hue, Saturation, Value (HSV; d–f), Lightness, Redness-greenness, and Yellowness-blueness (*L*a*b**; g–i). Trendlines represent samples collated from all groups (*n* = 320). Leafy vegetables within each category: Purple basil (PB), Red pak choi (RPC), Scarlet kale (SK), Greek basil (GB), Green pak choi (GPC), and Wasabi rocket (WR). FW, fresh weight; R^2, coefficient of determination (95% confidence interval).

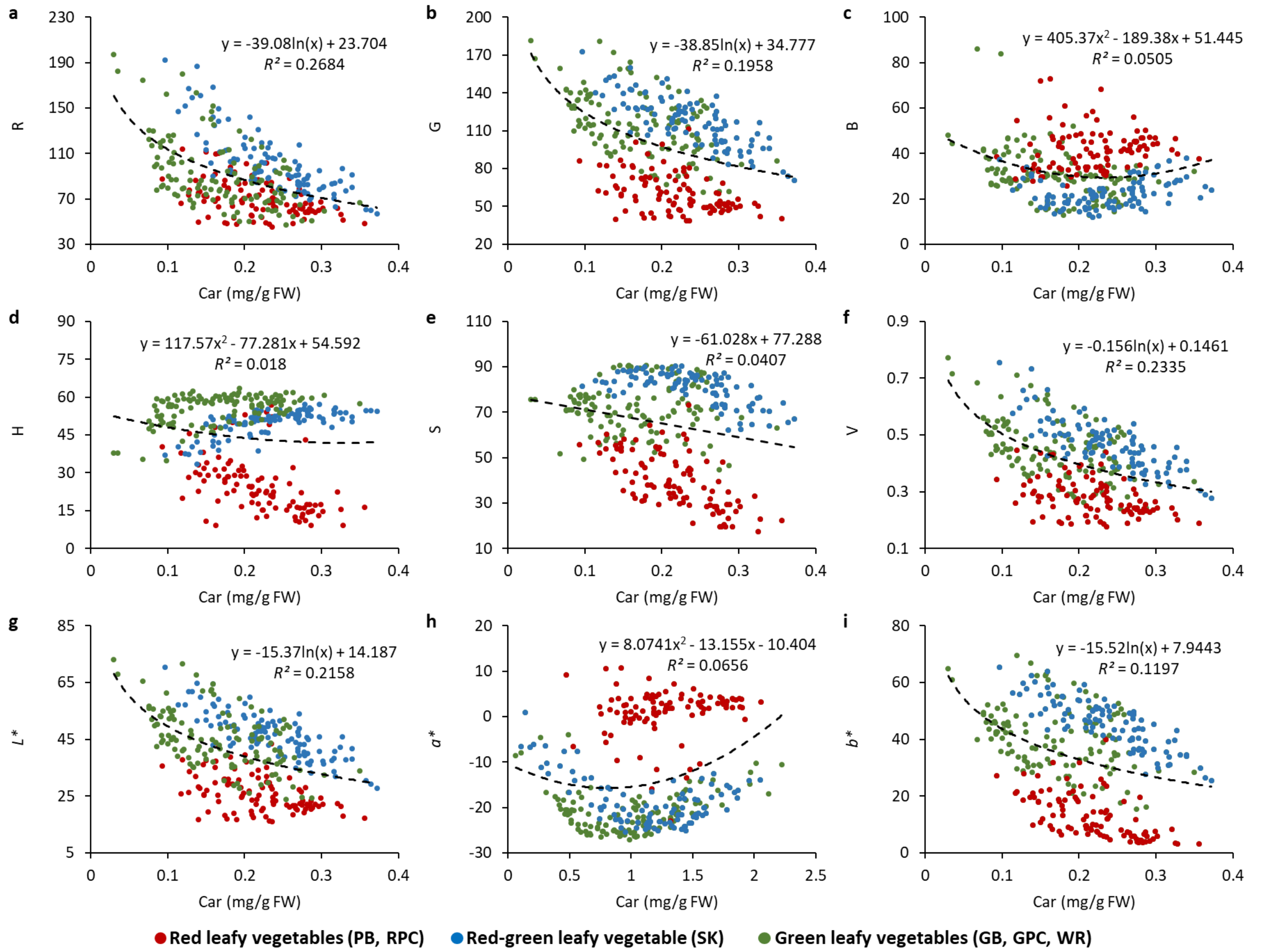

Fig. S2 Plots of leaf carotenoid (Car) content with different digital color features, i.e., Red, Green, Blue (RGB; a–c), Hue, Saturation, Value (HSV; d–f), Lightness, Redness-greenness, and Yellowness-blueness (*L* a*b**; g–i). Trendlines represent samples collated from all groups (n = 320). Leafy vegetables within each category: Purple basil (PB), Red pak choi (RPC), Scarlet kale (SK), Greek basil (GB), Green pak choi (GPC), and Wasabi rocket (WR). FW, fresh weight; R^2, coefficient of determination (95% confidence interval).

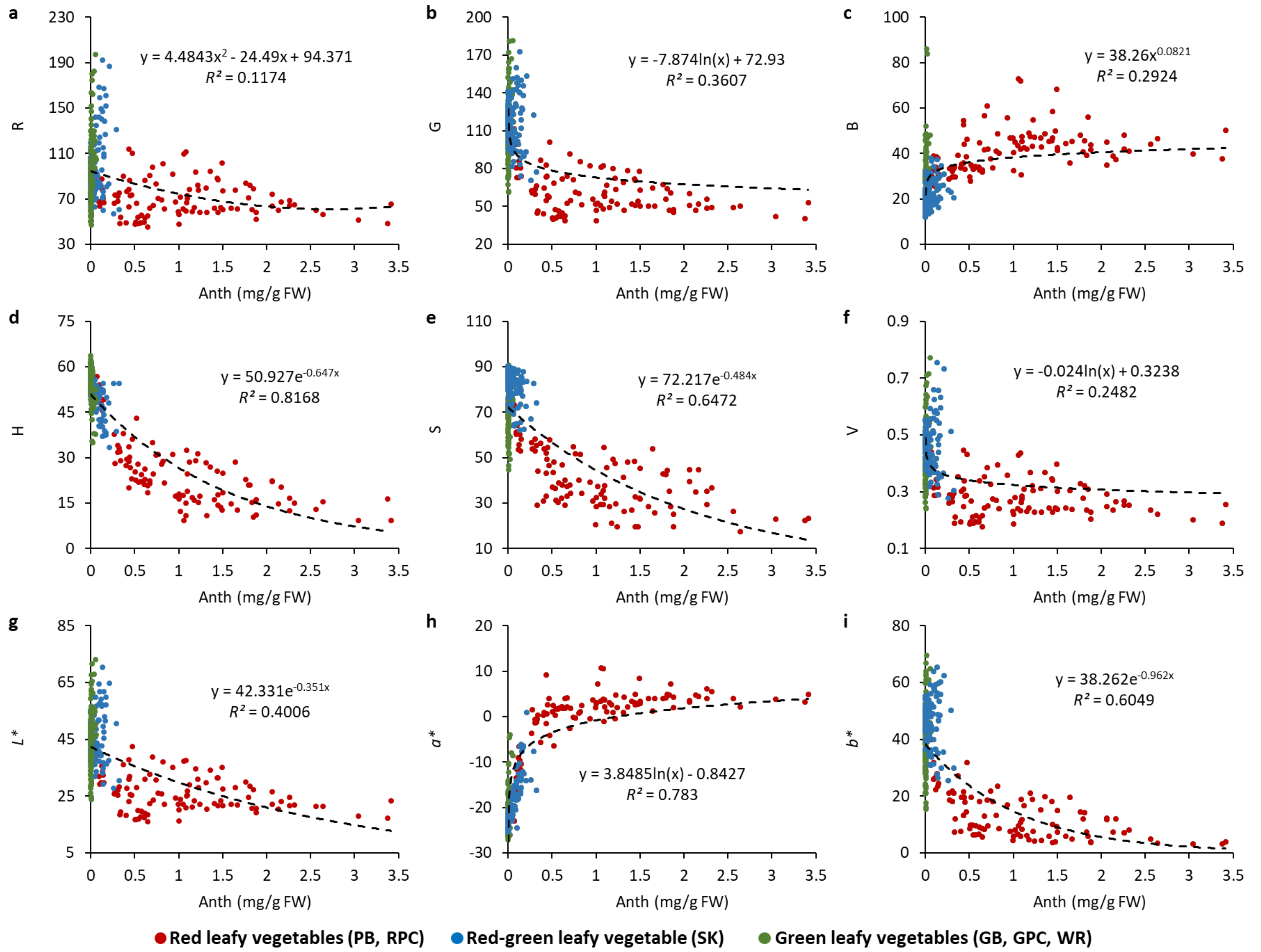

Fig. S3 Plots of leaf anthocyanin (Anth) content with different digital color features, i.e., Red, Green, Blue (RGB; a–c), Hue, Saturation, Value (HSV; d–f), Lightness, Redness-greenness, and Yellowness-blueness (*L* a*b**; g–i). Trendlines represent samples collated from all groups (*n* = 320). Leafy vegetables within each category: Purple basil (PB), Red pak choi (RPC), Scarlet kale (SK), Greek basil (GB), Green pak choi (GPC), and Wasabi rocket (WR). FW, fresh weight; R^2, coefficient of determination (95% confidence interval).

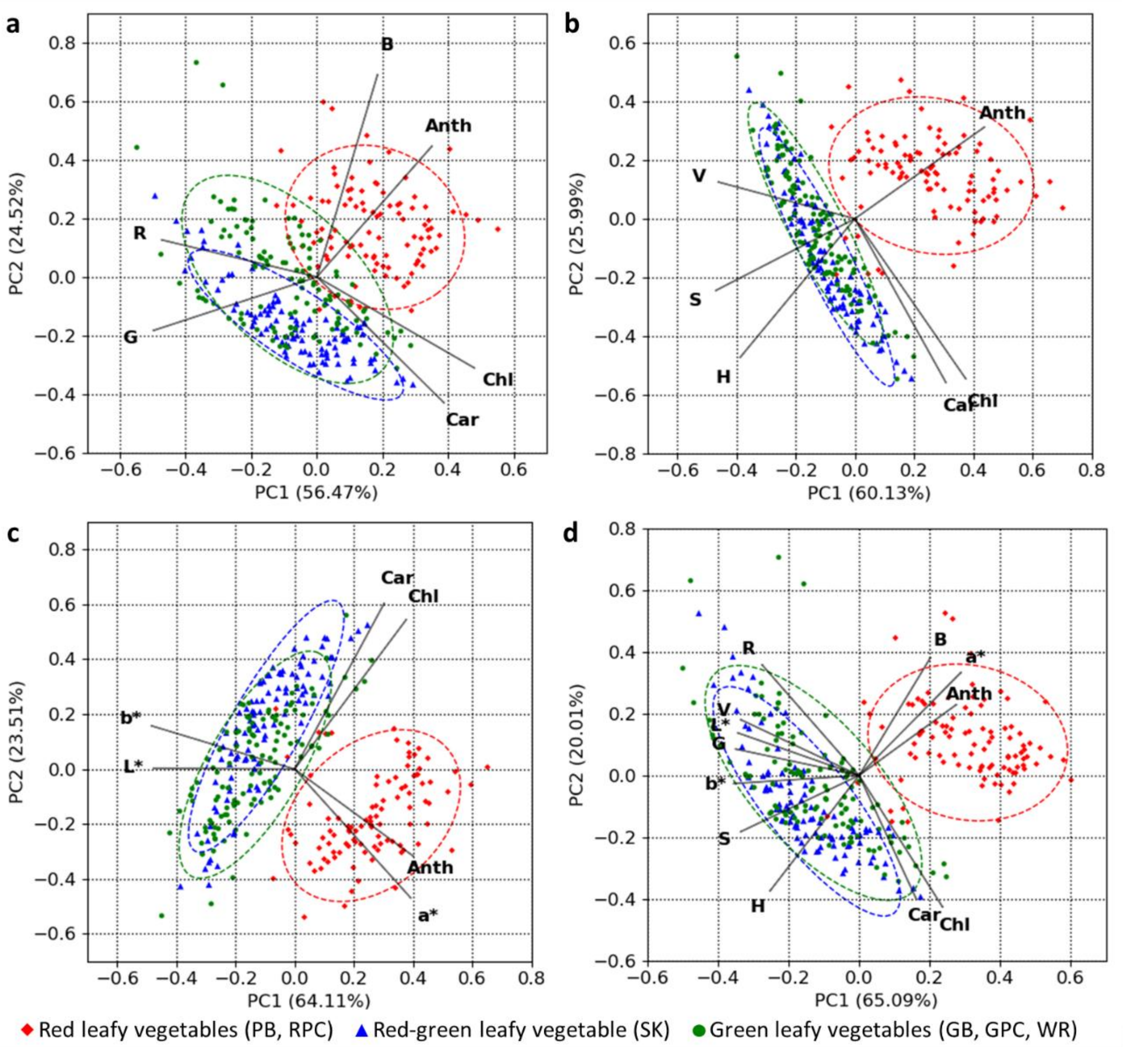

Fig. S4 PCA biplots depicting the relation of chlorophyll (Chl), carotenoid (Car), and anthocyanin (Anth) contents with the digital color profiles of leafy vegetables with varying visual profiles. Color space datasets: Red, Green, Blue (RGB; a); Hue, Saturation, Value (HSV; b); Lightness, Redness-greenness, Yellowness-blueness ($L^*a^*b^*$; c); all color spaces combined (d). PC1 and PC2, first and second principal components with values in parentheses indicating variance explained by each PC. Ellipses represent 95% confidence interval. Leafy vegetables within each category: Purple basil (PB), Red pak choi (RPC), Scarlet kale (SK), Greek basil (GB), Green pak choi (GPC), and Wasabi rocket (WR). Sample size: Green leafy vegetables, n = 120; Red-green leafy vegetable, n = 100; Red leafy vegetables, n = 100.

Supplementary tables

Table S1 Scaled Euclidean distance between the centroids (Δ*C*) of color feature-based scatter plots for the three categories of leafy vegetables.

**Plot variables/details**		**RLV-RGLV**	**RLV-GLV**	**RGLV-GLV**	**Reference**
Chlorophyll	R	0.217	0.199	0.096^	Fig. S1a
	G	0.405	0.404	0.074^	Fig. S1b
	B	0.272	0.205	0.135	Fig. S1c
	H	0.472	0.581#	0.121	Fig. S1d
	S	0.567#	0.463	0.141	Fig. S1e
	V	0.323	0.322	0.076^	Fig. S1f
	*L**	0.365	0.358	0.077^	Fig. S1g
	*a**	0.528#	0.6#	0.093^	Fig. S1h
	*b**	0.498	0.438	0.106	Fig. S1i
Carotenoid	R	0.212	0.202	0.203	Fig. S2a
	G	0.402	0.405	0.194	Fig. S2b
	B	0.268	0.207	0.224	Fig. S2c
	H	0.469	0.582#	0.216	Fig. S2d
	S	0.565#	0.464	0.228	Fig. S2e
	V	0.319	0.324	0.194	Fig. S2f
	*L**	0.362	0.36	0.195	Fig. S2g
	*a**	0.526#	0.601#	0.202	Fig. S2h
	*b**	0.496	0.439	0.208	Fig. S2i
Anthocyanin	R	0.358	0.341	0.066^	Fig. S3a
	G	0.495	0.49	0.026^	Fig. S3b
	B	0.394	0.344	0.115	Fig. S3c
	H	0.551#	0.643#	0.099^	Fig. S3d
	S	0.635#	0.54#	0.122	Fig. S3e
	V	0.431	0.425	0.03^	Fig. S3f
	*L**	0.463	0.453	0.034^	Fig. S3g
	*a**	0.6#	0.661#	0.063^	Fig. S3h
	*b**	0.574#	0.518#	0.08^	Fig. S3i
PCA without pigment contents	RGB	0.413	0.315	0.108	Fig. 2a
	HSV	0.555#	0.536#	0.087^	Fig. 2b
	*L*a*b**	0.53#	0.522#	0.093^	Fig. 2c
	All_3	0.507#	0.461	0.052^	Fig. 2d
PCA with pigment contents	RGB	0.372	0.291	0.145	Fig. S4a
	HSV	0.405	0.385	0.111	Fig. S4b
	*L*a*b**	0.434	0.393	0.134	Fig. S4c
	All_3	0.448	0.414	0.059^	Fig. S4d

Sample categories: GLV, green leafy vegetables (n = 120); RGLV, red-green leafy vegetables (n = 100); RLV, red leafy vegetables (n = 100). Color features: RGB, Red, Green, Blue; HSV, Hue, Saturation, Value; *L*a*b**, Lightness, Redness-greenness, Yellowness-blueness; All_3, combined dataset of RGB, HSV, and *L*a*b** color spaces. Lower values indicate greater overlap between sample classes, and vice versa. Δ*C* = 0 indicates perfect overlap, and Δ*C* = 1 indicates maximum separation. ^High overlap (Δ*C* < 0.1). #Low overlap (Δ*C* > 0.5).

Table S2 Relative importance of digital color features for estimating chlorophyll (Chl), carotenoid (Car), and anthocyanin (Anth) contents using different models.

Pigment	Feature	PLSR	SVR_*Lin*	SVR_*Pol*	SVR_*Rbf*	RFR_*5*	RFR_*10*	RFR_*50*
Chl	R	0.271	0.292	0.237	0.382	0.577	0.684	0.701
	G	0.446	0.121	0.099	0.071	0.011	0.017	0.011
	B	0.002	0.014	0.04	0.031	0.051	0.034	0.03
	H	<0.001	0.063	0.015	0.055	0.1	0.085	0.083
	S	<0.001	0.033	0.176	0.025	0.075	0.055	0.057
	V	0.001	0.221	0.156	0.16	0.045	0.022	0.022
	*L**	0.008	0.162	0.131	0.116	0.028	0.012	0.011
	*a**	0.213	0.007	0.008	0.091	0.054	0.057	0.062
	*b**	0.058	0.086	0.138	0.068	0.059	0.032	0.022
Car	R	0.239	0.013	0.207	0.063	0.073	0.122	0.141
	G	0.485	0.083	0.143	0.07	0.056	0.027	0.041
	B	0.002	0.165	0.031	0.254	0.237	0.265	0.239
	H	0.001	0.22	0.045	0.105	0.35	0.186	0.19
	S	<0.001	0.002	0.09	0.203	0.043	0.112	0.126
	V	<0.001	0.408	0.17	0.065	0.087	0.119	0.117
	*L**	0.028	0.088	0.157	0.068	0.052	0.035	0.036
	*a**	0.193	0.007	0.029	0.081	0.031	0.072	0.052
	*b**	0.053	0.015	0.127	0.089	0.071	0.061	0.057
Anth	R	0.043	0.204	0.016	0.017	0.03	0.009	0.009
	G	0.625	0.062	0.078	0.066	0.007	0.015	0.02
	B	0.001	0.002	0.074	0.1	0.034	0.056	0.062
	H	0.007	0.465	0.445	0.386	0.333	0.197	0.25
	S	0.004	<0.001	0.054	0.065	0.024	0.013	0.028
	V	0.004	0.06	0.055	0.024	0.004	0.003	0.006
	*L**	0.222	0.125	0.012	0.046	0.024	0.017	0.007
	*a**	0.084	0.011	0.194	0.206	0.445	0.653	0.531
	*b**	0.009	0.07	0.072	0.09	0.099	0.036	0.086

Color features: RGB, Red, Green, Blue; HSV, Hue, Saturation, Value; *L*a*b**, Lightness, Redness-greenness, Yellowness-blueness. Models: PLSR, Partial Least Squares Regression; SVR, Support Vector Regression with *linear* (*Lin*), *polynomial* (*Pol*), and *radial basis function* (*Rbf*) kernels; RFR_*n*, Random Forest Regression with *n* estimators. Three most important features for each model have been underlined. Each value indicates the mean of five model iterations generated using different random states. Each iteration involved ten repetitions of permutations.

Table S3 Equations and coefficients of determination (R^2) for linear correlation between actual chlorophyll (Chl) and carotenoid (Car) contents for the different leafy vegetables.

Plant	Equation	R^2	Samples (*n*)	Category
Purple basil	Car = 0.1292*Chl + 0.0609	0.832	60	Red leafy vegetable
Red pak choi	Car = 0.1231*Chl + 0.0439	0.688	40	
Scarlet kale	Car = 0.1284*Chl + 0.0882	0.893	100	Red-green leafy vegetable
Wasabi rocket	Car = 0.1047*Chl + 0.1137	0.815	40	Green leafy vegetable
Greek basil	Car = 0.1343*Chl + 0.0166	0.831	40	
Green pak choi	Car = 0.1139*Chl + 0.0284	0.927	40	
